# Severe Leptospirosis in Hospitalized Patients, Guadeloupe

**DOI:** 10.3201/eid1602.090139

**Published:** 2010-02

**Authors:** Cécile Herrmann-Storck, Magalie Saint Louis, Tania Foucand, Isabelle Lamaury, Jacqueline Deloumeaux, Guy Baranton, Maurice Simonetti, Natacha Sertour, Muriel Nicolas, Jacques Salin, Muriel Cornet

**Affiliations:** Guyane University, Guadeloupe, French West Indies (C. Herrman-Storck, M. Saint Louis, T. Foucand, I. Lamaury, J. Deloumeaux, M. Simonetti, M. Nicholas, J. Salin); Institut Pasteur, Paris, France (G. Baranton, N. Serour, M. Cornet); and Paris Descartes University, Paris (M. Cornet)

**Keywords:** Leptospirosis, prognostic factors, serovar, virulence, reservoir hosts, Guadeloupe, bacteria, dispatch

## Abstract

We evaluated prognostic factors for leptospirosis in 168 consecutive hospitalized patients in Guadeloupe. Factors independently associated with severity included chronic hypertension or chronic alcoholism, late initiation of antibacterial therapy, abnormal chest auscultation results, icterus, oligoanuria, disorders of consciousness, elevated aspartate aminotransferase levels, hyperamylasemia, and *Leptospira interrogans* serovar Icterohemorrhagiae.

Leptospirosis is a reemerging infectious disease in tropical and subtropical regions (*1*). In Guadeloupe, it has long been a major public health concern. Its incidence rate was ≈5.5/100,000 inhabitants per year from 1991 through 2002. Since 2003, this rate has greatly increased, peaking at 41.2/100,000 inhabitants in 2004 (*2*,*3*). The clinical features of leptospirosis vary and may progress to multiorgan failure and death (*4*). Initial clinical symptoms and laboratory test results associated with severe forms remain unclear. In this study, we focused on severe forms and determined prognostic factors that may help physicians in the early management of leptospirosis. We also characterized reservoir hosts by identifying the serovars of infecting strains. These findings will help establish appropriate control and prevention measures.

## The Study

This study was conducted in the hospital of Pointe-à-Pitre (1,100 beds), a tertiary referral center for Guadeloupe and neighboring islands. The ethical committee of the hospital approved the study. All consecutive patients hospitalized from January 2003 and through December 2004 with confirmed leptospirosis were included; patients admitted to Guadeloupe’s other hospital (200 beds) were excluded. Leptospirosis was confirmed if 1 blood culture yielded *Lesptospira* spp. or if specific antibodies were detected with either a single titer of >400 with the microscopic agglutination test (MAT) and an in-house enzyme immunoassay (EIA) with an immunoglobulin (Ig) M titer >400 (*5*) or at least a 4-fold increase in the MAT titer between the acute and convalescent phases. Cases were considered severe if dialysis (in case of oliguria) or mechanical ventilation was required or if the patient died. *Leptospira* serovars were isolated and identified as previously described (*3*). Epidemiologic, clinical, and laboratory data were collected retrospectively from medical records taken at patient’s admission. Data were analyzed by using Epi Info (Centers for Disease Control and Prevention, Atlanta, GA, USA). A multiple stepwise logistic regression analysis (SPSS, Chicago, IL, USA) was performed for variables with a p value <0.2.

During the 2-year period, leptospirosis was diagnosed in 168 hospitalized patients. A total of 132 case-patients had specific antibodies (49 had a single MAT titer >400 and EIA IgM titer >400, and 83 showed a 4-fold increase in the MAT titer in paired serum samples); 36 cases were confirmed only by culture. Of the 132 case-patients testing positive for antibody, 19 were also positive for bacterial culture. All but 2 case-patients were residents of Guadeloupe; the other 2 were a tourist from Paris and a resident of the Dominica. The ethnic distribution of the study population was similar to that of the Guadeloupean population.

We assessed patients’ demographic, epidemiologic, and clinical characteristics (Table 1). Twenty-four (14%) cases were considered to be severe: 6 (25%) of these were fatal. Female case-patients were significantly older than male case-patients (mean 58.5 ± 17.9 and 47 ± 15.9 years, respectively; p = 0.01). Chronic alcoholism was common (39%), especially among the 6 case-patients who died (67%). Chronic hypertension was also frequent (32%) (Table 1). The most common symptoms were myalgia (95%), headache (77%), digestive disorders (63%), fever (57%), abdominal pain (52%), and icterus (49%). Alveolar infiltrates was the most common feature, accounting for 9 (41%) of 22 anomalies observed in the lung by chest radiograph, followed by interstitial pattern (27%) and pleural suffusion (18%). Six case-patients with severe disease had cardiac complications: 2 had pericarditis confirmed by echocardiography, 2 had ischemic cardiac shock, and 2 myocardiopathy and myocarditis. Tomodensitometry or ultrasonography showed acute pancreatitis in 10 case-patients, of whom 6 had chronic alcoholism and 3 had severe disease. Thrombocytopenia (<150 × 10^9^ cells/L) was common (90% of case-patients), with severe thrombocytopenia (<50 × 10^9^ cells/L) observed in 19% of case-patients (Table 2). Hepatic cytolysis (alanine aminotransferase level >119 U/L or aspartate aminotransferase level >102 U/L) was found in 45% of case-patients. One fifth of case-patients exhibited rhabdomyolysis with creatinine phosphokinase levels >1,000 U/L (Table 2). The *L. interrogans* serovar Icterohemorrhagiae was found in 18 (45%) of the 40 case-patients for whom serovars were identified. The closely related *L. borgpetersenii* serovars Arborea and Castellonis accounted for 35% of identified strains (Table 2).

**Table 1:** Demographic, epidemiologic, and clinical characteristics as a function of severity among 168 case-patients with confirmed leptospirosis, Hospital of Pointe-à-Pitre, Guadeloupe, French West Indies, 2003–2004*

Characteristics	Univariate analysis†					Multivariate analysis	
	All case-patients, N = 168	Case-patients with severe disease, n = 24	Case-patients with nonsevere disease, n = 144	p value			
						OR (95% CI)	p value
Male sex	143/168 (85.1)	18/24 (75)	125/144 (86.8)	0.2		2.6 (0.4–17.7)	0.3
Age, y, mean ± SD (no. patients)							
F	58.5 ± 17.9 (25)	55.5 ± 2.4 (6)	36.7 ± 8 (19)	**0.05**			
M	47 ± 15.9 (143)	51.8 ± 6.5 (18)	49.6 ± 2.9 (125)	0.5			
Exposure to occupational risk‡	74/133 (55.6)	12/20 (60)	62 /113 (54.8)	0.6			
Contact with swine	41/115 (35.6)	5/10 (50)	36/105 (34.2)	0.5			
Contact with cattle	36/113 (31.9)	1/10 (10)	35/103 (34)	0.8			
Contact with rodents	56/114 (49.1)	3/9 (33.3)	53/105 (50.5)	0.5			
Medical history							
Duration of illness before antibacterial therapy§ >10 d	21/141 (14.9)	7/22 (31.8)	14/119 (11.8)	**0.01**		4.8 (1.1–20.2)	**0.032**
Diabetes mellitus	13/75 (17.3)	3/22 (13.6)	10/53 (18.9)	0.5			
Chronic hypertension ¶	24/75 (32)	9/22 (40.9)	15/53 (28.3)	0.2		30.9 (6.0–157.4)	**<0.001**
Chronic alcoholism#	29/75 (38.7)	11/22 (50.0)	18/53 (34)	0.2		16.8 (4.1–57.9)	**<0.001**
Initial features							
Hypothermia (<36.5°C)	28/144 (19.4)	7/23 (30.4)	21/121 (17.3)	0.1		4.6 (0.9–24.6)	0.07
Hyperthermia (>37.7°C)	82/144 (56.9)	10/23 (43.5)	72/121 (59.5)	0.1		3.8 (0.7–21.2)	0.12
Hypotension, SBP <100 mm Hg	24/143 (16.8)	7/20 (35)	17/123 (13.8)	**0.02**		0.3 (0.1–1.8)	0.2
Myalgia	73/77 (94.8)	12/13 (92.3)	61/64 (95.3)	0.6			
Consciousness disorders	10/119 (8.4)	4/13 (30.8)	6/106 (5.7)	**0.01**		3.8 (1.1–13.2)	**0.035**
Nuchal rigidity	10/116 (8.6)	2/12 (16.7)	8/104 (7.7)	0.2			
Headache	70/91 (76.9)	5/7 (71.4)	65/84 (77.4)	0.7			
Conjunctival suffusion	46/116 (39.6)	4/12 (33.3)	42/104 (40.4)	0.4			
Icterus	57/117 (48.7)	9/12 (75)	48/105 (45.7)	0.1		5.9 (1.1–31.1)	**0.036**
Hemorrhage**	15/165 (9.1)	5/24 (20.8)	10/141 (7.1)	**0.04**		4.2 (0.3–67.9)	0.31
Hepatosplenomegaly	30/86 (34.9)	7/11 (63.6)	23/75 (30.7)	**0.02**		1.7 (0.2–13.9)	0.62
Abdominal pain	64/124 (51.6)	14/18 (77.8)	50/106 (47.2)	**0.009**		3 (0.7–13.2)	0.139
Digestive disorders (diarrhea, vomiting)	66/105 (62.8)	12/15 (80)	54/90 (60)	0.2		3.5 (0.7–18.0)	0.12
Abnormalities at chest auscultation††	19/135 (14.1)	8/17 (47)	11/118 (9.3)	**<0.001**		8.7 (1.8–41.3)	**0.006**
Chest radiologic anomalies‡‡	22/112 (19.6)	6/16 (37.5)	16/96 (16.7)	**0.05**		0.8 (0.1–10.4)	0.85
Alveolar infiltrate	9/112 (8.0)	5/16 (31.2)	4/96 (4.2)	**<0.001**			
Electrocardiographic disorders§§	22/73 (30.1)	5/16 (31.2)	17/57 (29.8)	0.9			
Oliguria¶¶ or anuria	34/128 (26.6)	10/23 (43.5)	24/105 (22.9)	**0.04**		5.6 (1.5–20.6)	**0.009**

*OR, odds ratio; CI, confidence interval; SBP, systolic blood pressure. **Boldface** indicates significance. †All values no. case-patients/no. examined (%) except as indicated. ‡Farming, livestock farming, construction, and gardening. §Ampicillin or cefotaxime. ¶As reported by patients with specific therapy. #Defined as alcohol dependence. **Hemoptysis, hematuria, purpura, bleeding of the gums, and hematemesis. ††Crackles or ronchi. ‡‡Only anomalies of the lungs. §§Excluding patients >60 y of age or with sinus tachycardia. ¶¶Urinary volume <400 mL/d.

**Table 2:** Laboratory findings for 168 case-patients with confirmed leptospirosis, Hospital of Pointe-à-Pitre, Guadeloupe, French West Indies, 2003–2004*

Laboratory findings	Univariate analysis					Multivariate analysis	
	All case-patients, N = 168†	Case-patients with severe disease, n = 24†	Case-patients with nonsevere disease, n = 144†	p value			
						OR (95% CI)	p value
Prothrombin time <70%	10/118 (8.5)	5/21 (23.8)	5/97 (5.1)	**0.01**		0.7 (0.05–9.8)	0.077
Thrombocytopenia, <50 × 10^9^ cells/L	25/135 (18.5)	8/23 (34.8)	17/112 (15.2)	**0.02**		1 (0.1–2.4)	0.96
Hyperneutrophilia, >12 × 10^9^ cells/L	24/136 (17.6)	10/23 (43.5)	14/113 (12.4)	**0.01**		0.9 (0.1–7.1)	0.93
ALT >119 U/L	30/108 (27.8)	6/23 (26.1)	24/85 (28.2)	0.8			
AST >102 U/L	58/128 (45.3)	17/23 (73.9)	41/105 (39)	**0.02**		4.3 (1.2–14.6)	**0.021**
CPK >1000 U/L	22/108 (20.4)	5/18 (27.8)	17/90 (18.9)	0.4			
LDH >800 U/L	11/101 (10.9)	3/17 (17.6)	8/84 (9.5)	0.3			
Amylase >285 U/L	16/82 (19.5)	8/15 (53.3)	8/67 (11.9)	**<0.001**		18.5 (3.8–88.8)	**<0.001**
Lipase >60 U/L	7/40 (17.5)	2/8 (25)	5/32 (15.6)	0.8			
Hemoglobin, g/dL	12.9 ± 0.6 (136)	11.1 ± 2.0 (23)	13.2 ± 3.4 (113)	**0.01**			
Hemoglobin <10 g/dL	13/136 (9.5)	5/23 (21.7)	8/113 (7.1)	**0.04**		2.3 (0.5–9.8)	0.2
Bilirubin, mg/dL	8.3 ± 2.3 (86)	15.2 ± 11.7 (18)	5.8 ± 7.2 (68)	**<0.001**			
Bilirubin >7 mg/dL	31/86 (36.0)	13/18 (72.2)	18/68 (26.4)	**<0.001**		0.48 (0.05–4.7)	0.6
Potassium, mmol/L	4.0 ± 0.22 (136)	3.8±.9 (23)	4.0 ± 1.3 (113)	0.5			
Sodium, mmol/L	133.5 ± 4.2 (136)	132.8 ± 5.3 (23)	133.6 ± 3.9 (113)	0.5			
Creatinine, mg/d	2.0 ± 0.3 (130)	2.8 ± 2.5 (21)	1.9 ± 2.0 (109)	**0.05**		2.0 (0.6–6.6)	0.2
Creatinine >1.5 mg/dL	54/130 (41.5)	11/21 (52.4)	43/109 (39.4)	0.2			
Urea nitrogen mg/dL	69.0 ± 10.2 (136)	85.8 ± 76.2 (23)	64.2 ± 64.2 (113)	0.5			
Isolation of *Leptospira* in blood culture	55/88 (62.5)	8/14 (57.1)	47/74 (63.5)	0.3			
*L. interrogans* serovar Icterohemorrhagiae	18/40‡ (45)	6/8 (75)	12/32 (37.5)	0.06		5.3 (1.0–26.0)	**0.004**
*L. borgpetersenii* serovar Castellonis	5/40‡ (12.5)	0/8	5/32 (15.6)	0.1			
*L. borgpetersenii* serovar Arborea	9/40‡ (22.5)	2/8 (25)	7/32 (21.9)	0.6			
*L. kirschneri* serovar Bogvere	6/40‡ (15)	0/8	6/32 (18.7)	0.1			
*L. santarosai* serovar Tabaquite	2/40‡ (5)	0/8	2/8 (25)	0.4			

*OR, odds ratio; CI, confidence interval; ALT, alanine aminotransferase; AST, aspartate aminotransferase; CPK, creatinine phosphokinase; LDH, lactate dehydrogenase. **Boldface** indicates significance. †Mean ± SD (examined) or no. case-patients/no. examined (%). ‡Serovar identification was completed for 40 of the 55 *Leptospira* strains isolated.

Univariate analysis showed that, after stratification for sex, severity was associated with age for women but not for men. Neither occupation (farming, livestock farming, construction, and gardening) nor contact with swine, cattle, or rodents was linked to severity (Table 1). Nine host-related factors (listed in order of decreasing odds ratio) remained independently associated with severity in the multivariate analysis: history of chronic hypertension, hyperamylasemia, history of chronic alcoholism, abnormalities at chest auscultation, oligoanuria, late initiation (>10 days after onset of symptoms) of antibacterial therapy, elevated aspartate aminotransferase levels, consciousness disorders, and icterus. Chronic alcoholism was linked to death (p<0.01). The *L. interrogans* serovar Icterohemorrhagiae was isolated in 75% of severe cases, but in only 38% of nonsevere cases, and was independently associated with severity.

## Conclusions

The potential correlation between disease severity and *Leptospira* serovar remains a matter of debate. The *L*. *interrogans* serovars Icterohemorrhagiae, Canicola, and Australis have been linked to severity and multiorgan failure (*6*–*8*), but other studies did not confirm any link between serovar and outcome (*9*–*11*). *L.* Icterohemorrhagiae was clearly linked to disease severity. Therefore, a diagnostic test specifically detecting this serovar at an early stage of disease could help in the management of leptospirosis in patients in Guadeloupe. We confirmed that the *L. borgpetersenii* serovars Arborea and Castellonis, rarely isolated elsewhere, are highly prevalent in Guadeloupe (*3*,*12*). Taken together, they are the second most prevalent serovars after Icterohemorrhagiae. Notably, the serogroup Ballum, comprising the serovars Arborea and Castellonis, also is one of the main serogroups associated with human infections in Barbados (*9*). The serovar Arborea has been associated with mice, particularly in Barbados (*1*,*13*). In Guadeloupe, this serovar has been isolated from mice and rats, and the serovars Icterohemorrhagiae and Bogvere have been isolated from rats (*3*; V. Michelle, pers. comm.). Thus, rodent populations may be the main source of *Leptospira* spp. in Guadeloupe. Further animal studies are needed to establish the nature of these *Leptospira* reservoirs.

Chronic hypertension has not previously been found to predict poor prognosis for leptospirosis. Here, we found it to be the strongest risk factor for severe disease. Whether patients with histories of chronic hypertension are especially susceptible remains to be confirmed.

In our series of patients, acute hepatitis and pancreatitis were severe complications of leptospirosis in those with chronic alcoholism; chronic alcoholism itself was an independent indicator of poor prognosis. These results are consistent with findings from other studies conducted in La Réunion, another French overseas territory, and in continental France (*14*,*15*).

Patients with chronic hypertension or chronic alcoholism, late initiation of antibacterial therapy, consciousness disorders, abnormal features at chest auscultation, oligoanuria, jaundice, hyperamylasemia, or high aspartate aminotransferase levels may benefit from early intensive and specific management. The predominance of the Icterohemorrhagiae serovar, linked to severe disease, and of the Arborea and the Castellonis serovars highlights the need for rodent control to reduce the effects of leptospirosis in Guadeloupe.
